# Health related quality of life of patients undergoing in-centre hemodialysis in Rwanda: a cross sectional study

**DOI:** 10.1186/s12882-022-02958-6

**Published:** 2022-10-27

**Authors:** Gloria Shumbusho, Celestin Hategeka, Marianne Vidler, Jules Kabahizi, Marla McKnight

**Affiliations:** 1 Department of Internal Medicine, College of Medicine and Health Sciences, University of Rwanda, Kigali, Rwanda; 2 Department of Global Health and Population, Harvard TH Chan School of Public Health, Boston, MA USA; 3 Department of Obstetrics and Gynecology, University of British Columbia, Vancouver, BC Canada; 4 Department of Internal Medicine, Rwanda Military Hospital, University of Rwanda, Kigali, Rwanda; 5 Department of Medicine, University of British Columbia, Vancouver, BC Canada; 6 Department of Medicine, Harvard Medical School, Boston, MA USA

**Keywords:** Health related quality of life, Patient reported outcomes measures, End stage kidney disease, In-centre hemodialysis, Noncommunicable disease, Rwanda

## Abstract

**Background:**

There are few studies assessing the quality of life of patients with chronic and end stage kidney disease in sub-Saharan Africa. We aimed to describe the health-related quality of life (HRQOL) of patients undergoing in-centre maintenance hemodialysis in Rwanda using the KDQOL™-36 and determine sociodemographic and clinical factors associated with their quality of life.

**Methods:**

We conducted a multicenter, cross-sectional study between September 2020 and July 2021. Patients over the age of 18 receiving maintenance in-centre hemodialysis for at least three months at the Rwandan tertiary hospitals were administered the KDQOL™-36 questionnaire to assess physical and mental health functioning, the effect, burden and symptoms and problem of kidney disease. Sociodemographic and clinical information was collected for all eligible patients. Using mixed effects linear regression models, we explored factors associated with overall KDQOL and its domains, while accounting for clustering of patients within hemodialysis centres.

**Results:**

Eighty-nine eligible patients were included in the study. The majority of participants were younger than 60 years old (69.7%), male (66.3%), had comorbidities (91%), and 71.6% were categorized as level 3 on a 4 tier in-country poverty scale. All participants had health insurance coverage, with 67.4% bearing no out of pocket payments for hemodialysis. The median (IQR) quality of life score was 45.1 (29.4) for overall HRQOL, 35.0 (17.9) for PCS and 41.7 (17.7) for MCS. Symptoms and problem of kidney disease, effect of kidney disease, and burden of kidney disease scored 58.3 (43.8), 56.3 (18.8) and 18.8 (37.5), respectively. A notable difference of KDQOL scores between hemodialysis centres was observed. Overall KDQOL was associated with male sex (adjusted ß coefficient [aß]: 8.5, 95% confidence interval [CI]: 2.8, 14.3); being employed (aß: 8.2, 95% CI: 2.2, 14.3); dialysis vintage of 13–24 months (aß: 10.5, 95% CI: 3.6, 17.6), hemoglobin of 10-11 g/dl (aß: 7.3, 95% CI: 0.7, 13.7) and comorbidities (e.g., ≥ 3 comorbidities vs. none) (aß: -29.8, 95% CI: -41.5, -18.3).

**Conclusion:**

Patients on in-centre hemodialysis in Rwanda have reduced KDQOL scores, particularly in the burden of kidney disease and physical composite summary domains. Higher overall KDQOL mean score was associated with male sex, being employed, and dialysis vintage of 13–24 months, hemoglobin of 10-11 g/dl and absence of comorbidities. The majority of patients receiving in-centre hemodialysis have higher socioeconomic status reflecting the social and financial constraints to access and maintain dialysis in resource limited settings.

## Background

The burden of kidney disease worldwide is substantial and poses significant challenges for governments responding to the health of their populations, particularly in low and middle income countries (LMIC) [1]. In middle and eastern Africa, access to renal replacement therapy (RRT) is estimated at 1–3% [2] and the outcome of dialysis patients is poor, and marked by premature mortality in the first year after dialysis initiation [3, 4]. A high mortality rate following initiation of dialysis may be related to late presentation to a nephrologist or kidney care center, affordability, lack of access to treatment for complications and poor education [4].

Rwanda is one of the smallest central African countries with an approximately 13 million population and only about 17.6% living in urban areas[5]. The gross domestic product per capita is approximately 820 US dollars[6]. Over 92% of Rwandans access health care using community based health insurance (CBHI) with premium contribution depending on the household’s socioeconomic levels, also named “Ubudehe categories” [7]. Patients in category 1, the poorest, are exempt from premiums; category 2, 3 and 4, reflecting progressively higher socioeconomic status, pay a fixed co-pay for health center and hospital visits[8]. Approximately 6% of the total population have additional civil servant health insurance and military medical insurance (MMI) [7, 8]. In addition, there are private health insurance schemes available for purchase, and funds that cover medical care for vulnerable groups, such as genocide victim funds (GVF).

Based on estimates from the World Health Organization, noncommunicable diseases (NCDs) including renal diseases were the predominant cause of mortality in Rwanda, accounting for 58% of the mortality burden since 2016 [9]. There are insufficient data on the prevalence of kidney diseases in Rwanda, however, kidney failure was among the top 10 leading causes of death from non-communicable diseases and injuries in Rwanda in 2016 [9, 10]. Hemodialysis is the predominant renal replacement therapy available in Rwanda, very few patients are currently receiving peritoneal dialysis and renal transplantation is not performed in the country. Patients access transplantation through Ministry of Health funded transplantation performed out of country or through out-of-pocket payments abroad [10].

Access to dialysis is limited by its cost, a shortage of specialized medical staff with training in nephrology and renal replacement therapy and geographic distribution of in-centre hemodialysis units [10, 11]. The annual cost of hemodialysis per patient in Rwanda ranges between $13,260 USD and $20,592 USD. CBHI covers hemodialysis for six weeks for patients with acute kidney injury (AKI) as defined by Kidney Disease: Improving Global Outcomes (KDIGO) [12] and does not cover costs associated with RRT for chronic kidney disease (CKD) [13]. Maintenance dialysis is covered by employer and private health insurances or special funds (GVF) at 85 to 100% of hemodialysis costs, thus, for the majority of Rwandans, there are substantial out of pocket costs and financial hardship associated with hemodialysis [10].

Specialized kidney care and dialysis centers are primarily located in urban areas in Rwanda, particularly Kigali City, however, the majority of Rwandans live in rural areas [5]. Currently, in-centre maintenance hemodialysis is available at three public, university affiliated tertiary referral centers—Kigali University Teaching Hospital (CHUK), Rwanda Military hospital (RMH) located in the capital city, Kigali, and Butare University Teaching Hospital (CHUB) in the southern province. King Faisal Hospital (KFH), which is a public-private quaternary hospital also located in Kigali, houses the fourth in-centre dialysis unit in Rwanda. Community based hemodialysis is provided by African Health Network, a private company with three units located at Kimihurura (Kigali), Rubavu and Rusizi (Western province) with relatively similar cost, insurance coverage and out of pocket expenses as in-centre hemodialysis [10].

Between 2014 and 2017, approximately 47% of hemodialysis patients died within four months of initiation of dialysis at CHUK [13, 14]. There are dialysis dependent and non-dialysis dependent factors that impact patient experience and outcomes beyond dialysis adequacy including socioeconomic status, age, comorbidities, vascular access, dialysis session frequency, and symptoms associated with dialysis [15–21].

To promote high-quality services in renal dialysis facilities, routine measurement of patient reported outcomes measures such health-related quality of life (HRQOL) is recommended. In the United States, these measurements are typically done four months after initiation of dialysis and at least every year [22, 23]. The Kidney Disease Quality of Life (KDQOL) instrument designed by RAND Health Care and validated by the National Kidney Foundation for patients with kidney diseases may provide a reasonable metric of quality of life of adult patients living with end stage kidney disease (ESKD) in resource limited settings [24–26]. In Africa, there are few studies that have assessed the HRQOL of patients with CKD, less again of dialysis patients [27, 28].

In this study, we aimed to determine the health-related quality of life of patients with ESKD undergoing in-centre maintenance hemodialysis in Rwanda, describe demographic and clinical features of those patients and establish factors associated with their quality of life. We hypothesized that sociodemographic factors affect the HRQOL of patients undergoing renal replacement via hemodialysis in Rwanda. This is the first study of HRQOL in patients living with kidney disease in Rwanda and will provide baseline data that can help inform improvement strategies for ESKD patients on hemodialysis in Rwanda and other settings with similar contexts.

## Methods

**Fig. 1:**
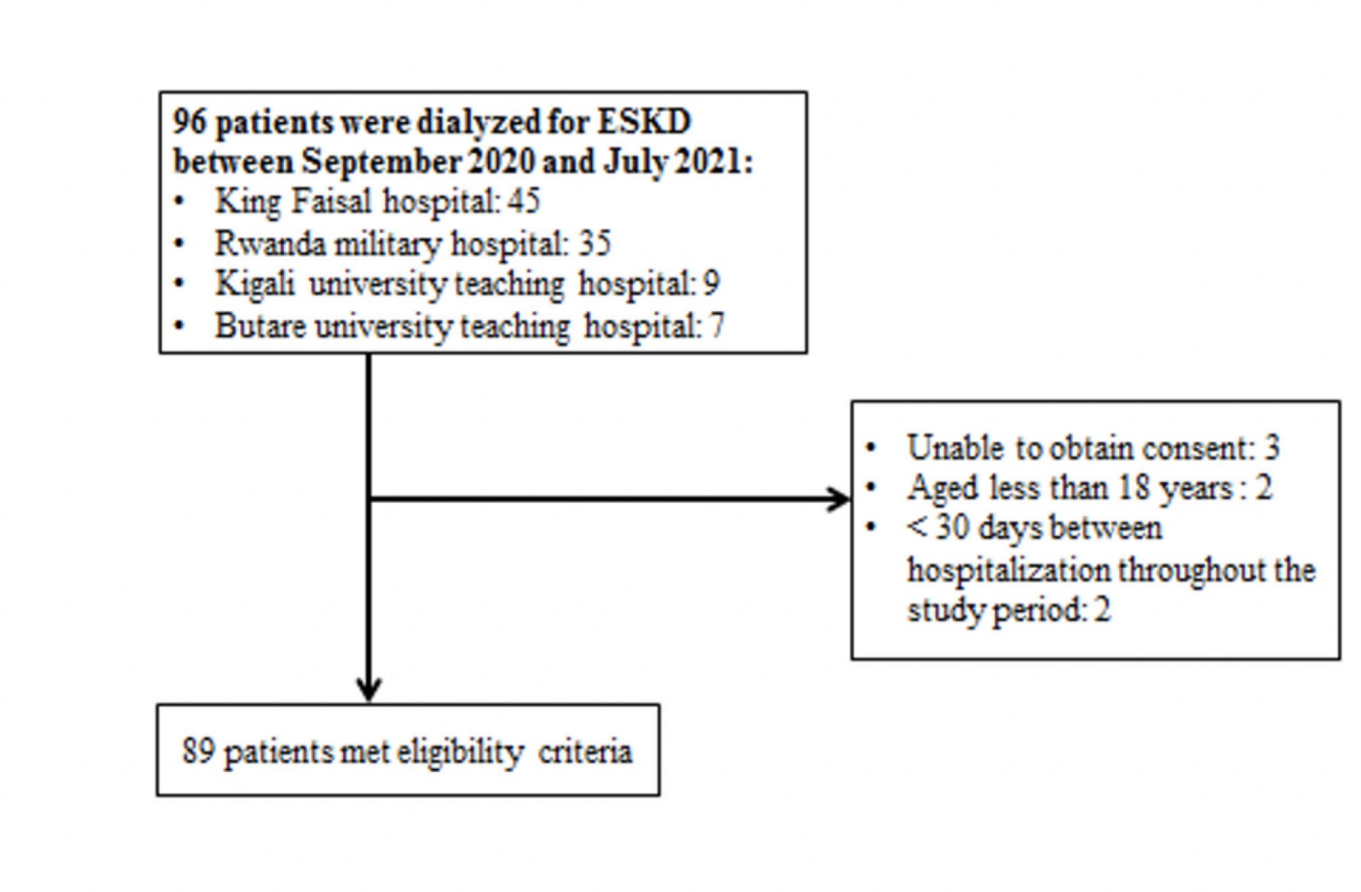
Patient inclusion in the study

We conducted a multicenter, prospective, cross-sectional study on all patients with ESKD disease undergoing in-centre hemodialysis in Rwanda between September 2020 and July 2021. In-centre hemodialysis units are located at Kigali University Teaching Hospital (CHUK), Rwanda Military hospital (RMH), Butare University Teaching Hospital (CHUB) and at King Faisal Hospital (KFH). Of the 96 patients on in-centre hemodialysis during the study period, we enrolled 89 (92.3%) of the adult patients (18 years and above) with ESKD on chronic in-centre maintenance hemodialysis (Fig. 1). We excluded patients who had undergone hemodialysis for less than 3 months to ensure patients met criteria for CKD and exclude patients with AKI as per KDIGO definitions. Patients hospitalized within the last four weeks and/or with neurological disability making them unable to respond to the questions were not included in our analysis. (Fig. 1)

### Data collection and measures

HRQOL data were collected using the KDQOL36-Item Short Form questionnaire. Scoring guides for the KDQOL™-36 from RAND Health Care were accessed and implemented. [24] Because of the relatively small number of participants at CHUK and CHUB, results from these two in-centre hemodialysis units were aggregated for analysis. To determine factors associated with HRQOL of hemodialysis patients, sociodemographic and clinical data were collected and all cutoffs were based on the distribution of the data. We did not collect data about small solute clearance using Kt/V as it was not measured in most dialysis centers. A questionnaire was administered to each participant during their regularly scheduled dialysis in person by one study investigator to ensure clarifying questions could be asked by the participant and ensure administration of the questionnaire was standardized. For patients not fluent in English, questions were translated by the study team into the local language. Informed consent was obtained from all participants in the study.

Responses from the quality of life questionnaire were exported to an excel scoring tool for the KDQOL™-36, which calculated patients’ scores in five domains (Physical composite summary PCS, Mental composite summary MCS, Burden of kidney disease BKD, Effect of kidney disease EKD and Symptoms and problem of kidney disease SPKD). Each item is scored from 0 to 100 representing the percentage of total possible score achieved. The higher score, the better the quality of life. [24] The overall KDQOL score was obtained from a programed KDQOL-36^™^ scoring tool [29]. All demographic and clinical information were collected on paper, entered into excel with independent double entry by two study investigators to minimize data entry errors.

### Analysis

Descriptive statistics were used to describe the study sample including demographic and clinical characteristics overall and by KDQOL. Mean and standard deviation or median with inter-quartile range (IQR) were used, as appropriate, for continuous variables and frequency with percentage for categorical variables. Mixed effects linear regression models were fitted to explore factors associated with overall KDQOL and its five domains, while accounting for clustering of patients within hemodialysis centers. First, unadjusted (crude) models were fitted to assess the association between each independent variable (e.g., sex, age, vintage, and comorbidity) and overall KDQOL (and its five domains) to check which variables pass an initial screening with α of 0.20 as model entry significance level. All potential factors associated with overall KDQOL (and its five domains) were retained for further exploration in multivariate (adjusted) models. Stepwise approach was used to select the most parsimonious models. Patients’ sex and age were retained in all models regardless of their α. Parameter estimates are reported as ß coefficients along with their 95% confidence interval (CI) and p values. All analyses were conducted using R version 4.0.2. Ethics approval was obtained through the University of Rwanda Institutional Review Board (IRB) Nº 053/CMHS IRB/2020 and the associated hospital sites.

## Results

### Demographic and clinical characteristics of study participants

The majority of participants were young with a male to female ratio of nearly 2:1. Almost all participants (91%) had comorbidities and 79.8% were taking > 3 medications per day. More than half (59.5%) had been on hemodialysis for more than 12 months with 80.9% were prescribed three times weekly hemodialysis. Among our study participants, 53.9% were married or living with their partner, 68.5% were living in urban areas and 40.4% had completed post-secondary education. All participants had health insurance coverage, with 67.4% covered at 100% with no out-of pocket costs. All participants in Ubudehe category 1 and 77.8% in Ubudehe category 2 had full coverage of costs, whereas in category 3 full coverage accounted for 60.3%. The majority of patients in the study (71.6%) were categorized in Ubudehe level 3. (Table 1)

### Health related quality of life

**Fig. 2:**
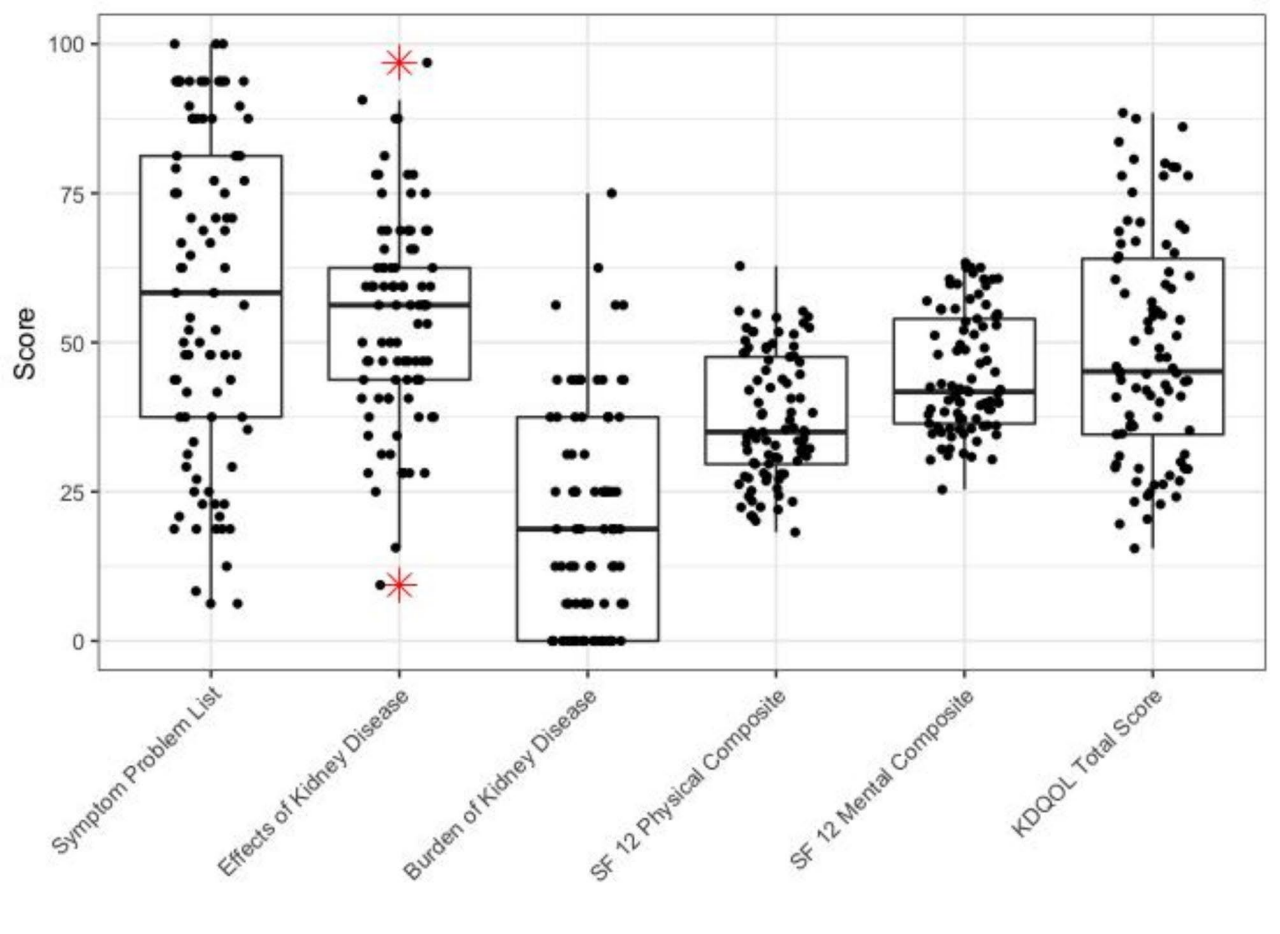
Distribution of HRQOL patients scores by KDQOL-36^™^ domains

The overall median quality of life score was 45.1 (IQR, 29.4). The physical composite summary (PCS) median score was 35.0 (IQR, 18.0) and mental composite summary (MCS) median score was 41.7 (IQR, 17.5). Symptoms and problems of kidney disease (SPKD) scored 58.33 (IQR, 43.8), whereas effect of kidney disease (EKD) and burden of kidney disease (BKD) had median (IQR) scores of 56.3 (18.8) and 18.8 (37.5) respectively (Fig. 2). Comparison between hemodialysis centers showed significant differences of HRQOL scores between hemodialysis centers and the overall KDQOL score SPKD, BKD, PCS and MCS scores (P value < 0.001). King Faisal Hospital’s hemodialysis unit had the highest HRQOL mean and median scores in all domains.

**Table 1:** Demographic and clinical characteristics of study participants

Variable	Category	Sample n = 89	%
**Sex**	Female	30	33.7
	Male	59	66.3
**Age**	< 45 years	29	32.6
	45–60 years	33	37.1
	> 60 years	27	30.3
**Education**	Post-secondary	36	40.4
	Secondary school	26	29.2
	Primary or less	27	30.3
**Marital status**	Married/ living together	48	53.9
	Never married	23	25.8
	Separated or widowed	18	20.2
**Ubudehe category**	1	7	8.0
	2	18	20.5
	3	63	71.6
	4	0	0
**Insurance coverage**	< 100% coverage	29	32.6
	100% coverage	60	67.4
**Hemodialysis centers**	CHUK-CHUB	14	15.7
	King Faisal Hospital	43	48.3
	Rwanda Military Hospital	32	36.0
**Employment status**	Employed	27	30.3
	Retired	12	13.5
	Unemployed	50	56.2
**Number of comorbidities**	0	8	9.0
	1	44	49.4
	2	28	31.5
	3 or more	9	10.1
**Number of medications**	< 3 drugs	18	20.2
	3–4 drugs	38	42.7
	> 4 drugs	33	37.1
**Hospitalized in the last 6 months (n = 87)**	No	40	46.0
	Yes	47	54.0
**Albumin g/l**	< 35	21	23.6
	35–40	40	44.9
	> 40	28	31.5
**Hemoglobin g/dl**	< 10	38	42.7
	10–11	24	27.0
	> 11	27	30.3
Prescribed number of HD sessions per week	2	17	19.1
	3	72	80.9
**Number of HD in past 30 days**	≤ 10 sessions	28	31.5
	> 10 sessions	61	68.5
**Hemodialysis access**	Fistula/ graft	30	33.7
	Semi-permanent dialysis catheter	34	38.2
	Temporary dialysis catheter	25	28.1
**Dialysis vintage**	< 12 months	36	40.4
	13–24 months	22	24.7
	> 24 months	31	34.8

#Ubudehe category: economic life standing of households of Rwandan population

Factors independently associated with health related quality of life.

In adjusted models, on average male participants had significantly higher HRQOL overall and in the physical composite summary and the symptoms and problems of kidney disease domains (Tables 2 and 3). Participants who were employed had significantly greater HRQOL overall and in the physical composite summary, mental composite summary, and the burden of kidney disease domains **(**Tables 2 and 3**).** Similarly, participants with no comorbidities had higher HRQOL overall and in all five domains **(**Tables 2 and 3**).** Likewise, participants who had had hemodialysis for more than one year had on average greater HRQOL overall and in the domains of mental composite summary and the symptoms and problems of kidney disease. Participants with hemoglobin level between 10 and 11 g/dl had significantly greater overall kidney disease quality of life; whereas, those with fistula for hemodialysis access had significantly higher HRQOL in the burden of kidney disease domain.

## Discussion

In the current study, we found patients undergoing in-centre hemodialysis demonstrate low quality of life scores. HRQOL results were lower across all domains in comparison to studies that have used the KDQOL-36™ tool in high income countries. Similar scores in the physical and mental component summary were seen in comparison to studies conducted in other LMIC, however, the symptom and problem of kidney disease scores in Rwanda were much lower than those seen Kenya and Saudia Arabia **(**Table 4**)** [28, 30–32]. While our findings cannot be directly compared to HRQOL studies using the KDQOL-SF questionnaire in similarly resourced low income settings in sub-Saharan Africa, our findings are broadly in alignment with studies undertaken in geographically and economically similar settings. Using the KDQOL-SF, Masina et al. in Malawi [27] and Bagasha et al. in Uganda [29] found an overall HRQOL score of 59.9 (± 8.8) and 41.71 (± 4.42) respectively. The burden of kidney disease had a lower score (20.01 ± 18.27) and symptoms and problem of kidney disease domain had relatively higher mean score (58.22 ± 27.44) more marked in patients above 60 (ß: 13.611; 95% CI: 1.42, 26.29). Similar findings of low scores in the burden of kidney disease sub-scale and relatively higher score in the symptoms and problem of kidney disease sub-scale have been noted in studies conducted in sub-Saharan Africa [27–29]. This suggests that the KDQOL-36 scoring system, which is freely available and shorter to administer than the KDQOL-SF, may be reasonably employed in settings with already constrained human and economic resources to assess an outcome that is important to patients undergoing hemodialysis.

Similar to studies of hemodialysis patients conducted in LMICs, 69.7% of participants were younger than 60 years of age reflecting the epidemiology of kidney disease observed in LMICs, the lack of strategies for prevention and management of communicable and non-communicable diseases, poor socioeconomic status and the limited access to dialysis and transplantation [10, 20, 27–29, 33–35]. There was a positive association between dialysis vintage and higher QoL scores, which may reflect survivorship bias and adaptation of patients dialyzed for a longer period but prior to developing complications of longer term dialysis. [16, 20, 28, 35, 36] In this study, the number of comorbidities was in direct proportion with worse quality of life score, affecting all HRQOL domains, which is similar to a study conducted by Cha et al. demonstrating significantly poor HRQOL associated with the high number of comorbidities in Korea (p < 0.001) [19].

Our results showed higher overall KDQOL score and in all five domains in patients on in-centre hemodialysis at King Faisal Hospital and lower scores at the CHUK-CHUB in-centre hemodialysis units. KFH is the most accredited hospital of the country, was the first hemodialysis unit in Kigali with more beds than other centers, specialized medical staff, greater human resources and materials. In addition, the national referral board office that transfers patients for kidney transplantation is located at KFH and patients access hemodialysis using health insurance coverage as in other in-centre dialysis units. While RRT care is covered by insurance similarly at all in-centre sites, additional care—consultations, investigations, admission to hospital—often is associated with additional out of pocket costs than would be borne at the public hospitals. The majority of patients managed at CHUK and CHUB are referred from rural areas with lower socioeconomic status with fewer patients on chronic maintenance hemodialysis at those units. The improved HRQOL of patients undergoing hemodialysis at KFH likely reflects the experience and resources available at KFH as well as the ability to access care at the in-centre site of patient preference.

In our study, most patients were living in Kigali where three of four in-centre hemodialysis units are located. Patients and their family are required to travel long distances or relocate near dialysis centers to undergo dialysis and this in turn affects their daily activities and relationships with others, which likely contributed to reduced burden of kidney disease domain scores. Many patients had obtained a relatively higher level of education and were from a relatively higher socioeconomic status in ubudehe category 3, echoing other African studies that have highlighted that the majority of patients on hemodialysis are largely from a higher socioeconomic status [10, 28, 29, 35–37]. Interestingly, there were no patients in the highest ubudehe category on in-centre hemodialysis in Rwanda. This finding was corroborated with the African Health Network community dialysis units where there are also few patients in ubudehe category 4 receiving hemodialysis (personal communication from AHN health care provoder, August 2021). It is possible that patients with the highest socioeconomic status in Rwanda access care early with fewer progressing to ESKD and those who do progress relocate out of country to access RRT and transplantation. Further, our study also demonstrated a lower number of patients in ubudehe categories 1 and 2 undergoing in-centre hemodialysis, which likely reflects the barrier of out of pocket costs associated with RRT. These findings reflect the financial constraints and the social impact to access and maintain RRT in resource limited settings.

### Study limitations:

There are a number of limitations that need to be acknowledged. First, while the KDQOL-36^™^ instrument has been validated in other contexts, it is yet to be validated in Rwanda and there is no validated translation into the local language (Kinyarwanda). Therefore, it is possible there could have been misinterpretations by study participants related to translation of the questionnaire and local validation of the KDQOL survey instrument in future studies is recommended. Second, being a cross-sectional study we are unable to infer causality of low HRQOL scores. Third, while the sample size appears relatively small, we included all eligible patients accessing in-centre hemodialysis. Fourth, our study did not include data from the three community dialysis units in the country, thus our results may not be generalizable to the overall hemodialysis population in Rwanda. Lastly, while our study does demonstrate the impact of socioeconomic status on HRQOL, our analysis could have been strengthened by the inclusion of additional variables. Future studies could include metrics exploring socioeconomic factors such as relocation or travel time to dialysis centres, out of pocket costs, yearly income or use of a validated poverty index to further evaluate the impact of poverty on quality of life.

## Conclusion

Patients on in-centre hemodialysis in Rwanda have low HRQOL scores. The lowest score was found on burden of kidney disease and physical composite summary domains and there is a notable difference of HRQOL scores between hemodialysis units in Rwanda. Factors associated with overall HRQOL found were sex, employment status, number of comorbidities, dialysis vintage, and hemoglobin level, thus, optimizing medical and biomedical management of dialysis patients and finding ways to make dialysis less obstructive to maintain employment may assist in improving HRQOL. Further studies on HRQOL in both in-centre and community-based units as well as comparisons between using internationally accepted measures of dialysis adequacy (Kt/V) and QOL measures which are cheap and easy to administer in low income countries are recommended. Most patients in Rwanda on hemodialysis have higher socioeconomic status reflecting the financial constraints and the social impact to access and maintain renal replacement therapy in resource limited settings. As such, improving equitable access to RRT should remain a priority.

**Table 2:** Factors associated with overall KDQOL, PCS and MCS

Independent variables (reference group)		Overall KDQOL				Physical.Composite Summary				Mental.Composite Summary				
		**Crude ß coefficient (95% CI)**	**P value**	**Adjusted ß coefficient (95% CI)**	**P value**	**Crude ß coefficient (95% CI)**	**P value**	**Adjusted ß coefficient (95% CI)**	**P value**	**Crude ß coefficient (95% CI)**	**P value**	**Adjusted ß coefficient (95% CI)**		**P value**
**Sex (Female)**														
**Male**	6.57 (0.02, 13.04)		0.04	8.54 (2.77, 14.26)	0.008	6.84 (3.24, 10.41)	< 0.001	4.62 (1.02, 8.21)	0.01	-0.45 (-4.39, 3.42)	0.81		0.61 (-2.86, 4.12)	0.74
**Age (< 45 years)**														
**45–60 years**	3.35 (-4.32, 11.49)		0.40	6.23 (-0.37, 13.14)	0.09	-2.76 (-7.19, 1.91)	0.23	-1.32 (-5.40, 2.96)	0.55	4.04 (-0.37, 8.85)	0.08		3.83 (-0.17, 8.39)	0.08
**> 60 years**	5.37 (-2.43, 13.47)		0.18	6.68 (-1.67, 15.17)	0.15	-3.92 (-8.42, 0.74)	0.09	-4.19 (-9.50, 1.20)	0.14	4.56 (0.05, 9.31)	0.05		1.09 (-3.86, 6.36)	0.68
**Education (Primary school and less)**														
**Secondary school**	2.38 (-6.06, 10.56)		0.56			0.75 (-4.16, 5.52)	0.75			2.58 (-2.24, 7.45)	0.24			
**Post-secondary**	2.17 (-5.33, 9.57)		0.57			0.62 (-3.76, 4.93)	0.77			2.70 (-1.81, 6.89)	0.27			
**Marital status (Never married)**														
**Married/living together**	3.59 (-3.63, 10.89)		0.33			-1.09 (-5.17, 3.01)	0.60			1.37 (-2.94, 5.75)	0.53			
**Separated/widowed**	-4.77 (-13.75, 4.31)		0.30			-7.61 (-12.67, -2.49)	0.004			-1.48 (-6.84, 3.96)	0.59			
**Employment (Unemployed)**														
**Employed**	5.99 (-0.85, 12.89)		0.09	8.16 (2.18, 14.29)	0.01	4.85 (0.92, 8.80)	0.01	3.88 (0.39, 7.39)	0.04	2.32 (-1.66, 6.34)	0.25		5.76 (2.09, 9.53)	0.005
**Retired**	6.93 (-2.32, 16.37)		0.14	4.23 (-5.01, 13.70)	0.41	4.18 (-1.12, 9.58)	0.12	5.68 (0.05, 11.40)	0.06	6.45 (1.07, 11.96)	0.02		8.18 (2.66, 13.86)	0.007
**Ubudehe category (1)**														
**2**	1.78 (-10.99, 14.66)		0.78			-0.75 (-7.75, 6.30)	0.83			-2.87 (-10.46, 4.80)	0.46			
**3**	6.84 (-4.62, 18.56)		0.25			4.41 (-1.87, 10.83)	0.17			-1.04 (-7.84, 5.96)	0.76			
**Health insurance coverage (< 100% coverage)**														
100% coverage	4.77 (-1.84, 11.28)		0.15			0.34 (-3.55, 4.17)	0.86			1.08 (-2.86, 4.93)	0.58			
**Prescribed number of HD sessions per week (Twice)**														
**Thrice**	-1.47 (-9.67, 6.46)		0.71			-0.48 (-5.26, 4.13)	0.83			-0.30 (-5.15, 4.35)	0.89			
**Number of HD in the past 30 days (≤ 10)**														
**> 10**	-2.53 (-9.19, 4.09)		0.45			-0.39 (-4.27, 3.47)	0.84			-0.15 (-4.08, 3.74)	0.93			
**Hospitalized in the last 6 months (No)**														
**Yes**	-3.61 (-9.88, 2.63)		0.25			-4.68 (-8.22, -1.15)	0.01			-1.97 (-5.66, 1.68)	0.29			
**Number of medications taking (< 3 drugs)**														
**3–4 drugs**	-2.32 (-10.76, 6.01)		0.58			-4.22 (-9.06, 0.54)	0.08			-2.75 (-7.67, 2.08)	0.27			
**> 4 drugs**	-2.74 (-11.48, 5.80)		0.53			-3.72 (-8.74, 1.16)	0.14			-0.12 (-5.23, 4.83)	0.96			
**Albumin (< 35 g/l)**														
**35–40 g/l**	8.36 (0.65, 16.27)		0.03	6.29 (-0.39, 13.17)	0.09	3.07 (-1.47, 7.76)	0.19			1.97 (-2.65, 6.77)	0.41			
**> 40 g/l**	8.86 (0.66, 17.27)		0.03	2.47 (-4.92, 10.01)	0.54	3.76 (-1.07, 8.75)	0.13			2.36 (-2.55, 7.46)	0.35			
**Hemoglobin (< 10 g/dl)**														
**10–11 g/dl**	9.44 (2.03, 16.82)		0.01	7.27 (0.70, 13.72)	0.04	1.84 (-2.59, 6.26)	0.41			5.05 (0.66, 9.40)	0.02			
**> 11 g/dl**	2.39 (-4.75, 9.59)		0.51	2.09 (-3.94, 8.13)	0.53	-0.59 (-4.89, 3.71)	0.78			2.65 (-1.58, 6.91)	0.22			
**Vintage (≤ 12 months)**														
**13–24 months**	8.65 (0.83, 16.80)		0.03	10.47 (3.57, 17.58)	0.008	-0.44 (-5.09, 4.40)	0.85			7.48 (3.04, 12.19)	0.001		9.10 (5.03, 13.46)	< 0.001
**> 24 months**	5.70 (-1.36, 13.03)		0.12	9.71 (3.33, 16.30)	0.007	1.52 (-2.68, 5.87)	0.48			3.40 (-0.6, 7.63)	0.107		6.30 (2.54, 10.30)	0.003
**HD access (Fistula/graft)**														
**Semi-permanent dialysis catheter**	-8.44 (-16.35, -0.89)		0.03			-6.20 (-10.69, -1.90)	0.006	-6.11 (-10.38, -1.94)	0.008	-3.88 (-8.62, 0.57)	0.09			
**Temporary dialysis catheter**	-6.75 (-14.71, 1.01)		0.09			-5.59 (-10.12, -1.17)	0.01	-5.30 (-9.51, -1.16)	0.02	-3.00 (-7.75, 1.59)	0.21			
**Number of comorbidities (0)** ^**$**^														
**1**	-11.40 (-22.62, -0.52)		0.04	-15.31 (-25.17, -5.76)	0.005	-0.12 (-6.52, 6.08)	0.96	-3.28 (-9.11, 2.40)	0.29	-10.04 (-16.60, -3.74)	0.003		-9.55 (15.58, -3.82)	0.003
**2**	-11.14 (-22.79, 0.13)		0.06	-13.20 (-23.30, -3.46)	0.01	-4.55 (-11.20, 1.88)	0.17	-3.36 (-9.64, 2.72)	0.31	-6.51 (-13.33, 0.021)	0.059		-6.51 (-12.96, -0.57)	0.04
**3 or more**	-21.49 (35.22, -8.03)		0.002	-29.97 (-41.47, -18.32)	< 0.001	-9.49 (-17.33, -1.81)	0.01	-9.06 (-16.42, -1.84)	0.02	-10.81 (-18.84, -3.012)	0.009		-12.55 (-19.97, -5.60)	0.001

*Adjusted for clustering of patients within hemodialysis centers

^$^ Comorbidities include hypertension, heart failure, hepatitis B or C, cerebrovascular disease, HIV/AIDS and gout

**Table 3:** Factors associated with BKD, EKD and SPKD domains

IndepenIndependent variables (reference) group)	Burden of kidney disease				Effects of kidney disease				Symptom problem of kidney disease			
	**Crude ß coefficient (95% CI)**	**P value**	**Adjusted ß coefficient (95% CI)**	**P value**	**Crude ß coefficient (95% CI)**	**P value**	**Adjusted ß coefficient (95% CI)**	**P value**	**Crude ß coefficient (95% CI)**	**P value**	**Adjusted ß coefficient (95% CI)**	**P value**
**Sex (Female)**												
**Male**	7.17 (-0.11, 14.32)	0.05	2.95 (-4.49, 10.29)	0.46	6.51 (-1.27, 13.78)	0.09	6.84 (-0.35, 14.03)	0.07	5.51 (-4.75, 15.66)	0.29	11.98 (2.82, 21.18)	0.01
**Age (< 45 years)**												
**45–60 years**	-0.71 (-9.23, 8.55)	0.87	1.16 (-7.12, 10.49)	0.80	2.25 (-6.31, 10.82)	0.61	7.24 (-0.98, 15.47)	0.10	7.30 (-4.46, 19.91)	0.23	11.49 (0.50, 23.35)	0.05
**> 60 years**	0.99 (-7.68, 10.15)	0.82	-1.70 (-12.81, 9.70)	0.78	3.02 (-5.98, 12.02)	0.51	9.09 (-0.42, 18.61)	0.07	9.87 (-2.10, 22.39)	0.11	13.611 (1.42, 26.29)	0.04
**Education (Primary school and less)**												
**Secondary school**	0.11 (-9.22, 9.02)	0.98			0.51 (-8.69, 9.44)	0.91			3.75 (-9.30, 16.34)	0.87		
**Post-secondary**	5.68 (-2.58, 13.77)	0.17			7.51 (-0.93, 15.87)	0.08			-0.94 (-12.55, 10.45)	0.56		
**Marital status (Never married)**												
**Married/living together**	0.17 (-7.86, 8.32)	0.96			0.04 (-8.49, 8.62)	0.99			5.81 (-5.53, 17.27)	0.32		
**Separated/widowed**	-8.12 (-18.10, 2.02)	0.11			1.52 (-9.05, 12.18)	0.78			-0.87 (-14.96, 13.41)	0.90		
**Employment (Unemployed)**												
**Employed**	9.78 (2.34, 17.31)	0.01	10.29 (3.20, 17.49)	0.009	12.35 (4.72, 19.98)	0.002			-0.12 (10.91, 10.76)	0.98		
**Retired** **8.12 (-1.92, 18.43)**		0.12	10.57 (-0.96, 22.57)	0.10	6.33 (-3.93, 16.60)	0.23			4.01 (-10.56, 18.91)	0.59		
**Ubudehe category (1)**												
**2**	8.34 (-5.78, 22.63)	0.25			3.51 (-11.22, 18.52)	0.64			8.71 (-11.00, 28.62)	0.39		
**3**	7.09 (-5.54, 20.15)	0.28			3.06 (-9.93, 16.67)	0.65			6.89 (-10.77, 25.05)	0.45		
**Health insurance coverage (< 100% coverage)**												
**100% coverage**	1.60 (-5.85, 8.88)	0.66			-2.18 (-9.81, 5.42)	0.57			13.03 (3.06, 22.81)	0.01	9.57 (0.01, 18.74)	0.05
**Prescribed number of HD sessions per week (twice)**												
**Thrice**	-0.56 (-9.74, 8.20)	0.90			-11.83 (-20.59, -3.07)	0.009			2.21 (-10.46, 14.46)	0.72		
**Number of HD in the past 30 days (≤ 10)**												
**> 10**	-1.34 (-8.75, 6.02)	0.72			-9.08 (-16.56, -1.62)	0.01	-7.70 (-14.81, -0.59)	0.04	-1.91 (-12.22, 8.34)	0.71		
**Hospitalized in the last 6 months (No)**												
**Yes**	-3.73 (-10.62, 3.11)	0.28			-8.30 (-15.42, -1.15)	0.025			0.75 (-9.08, 10.53)	0.87		
**Number of medications taking (< 3 drugs)**												
**3–4 drugs**	-5.10 (-14.33, 3.96)	0.27			-5.69 (-15.26, 3.87)	0.25			-7.48 (-20.31, 5.16)	0.25		
**> 4 drugs**	1.64 (-7.95, 10.92)	0.73			-6.07 (-15.87, 3.72)	0.23			-12.43 (-25.73, 0.52)	0.06		
**Albumin (< 35 g/l)**												
**35–40 g/l**	7.24 (-1.29, 16.14)	0.10			2.19 (-6.89,11.27)	0.64			-1.02 (-13.24, 11.61)	0.87		
**> 40 g/l**	9.62 (0.51, 19.08)	0.04			2.71 (-7.01, 12.44)	0.58			0.36 (-12.65, 13.77)	0.95		
**Hemoglobin (< 10 g/dl)**												
**10–11 g/dl**	9.03 (0.74, 17.25)	0.03			4.80 (-3.93, 13.54)	0.28			11.12 (-0.50, 22.68)	0.06		
**> 11 g/dl**	1.17 (-6.81, 9.21)	0.77			2.84 (-5.58, 11.29)	0.51			6.05 (-5.16, 17.34)	0.29		
**Vintage (≤ 12 months)**												
**13–24 months**	5.10 (-3.66, 14.44)	0.26			4.82 (-4.16, 13.88)	0.30			18.70 (6.97, 31.03)	0.002	16.28 (5.20, 27.82)	0.008
**> 24 months**	-0.01 (-7.95, 8.35)	0.99			-2.76 (-10.89, 5.44)	0.51			11.61 (1.001, 22.68)	0.03	9.74 (-0.35, 20.31)	0.07
**HD access (Fistula/graft)**												
**Semipermanent dialysis catheter**	-8.59 (-17.38, -0.37)	0.04	-8.01 (-16.95, 0.51)	0.09	-2.87 (-11.30, 5.64)	0.50			-8.89 (-21.34, 2.92)	0.15		
**Temporary dialysis catheter**	-10.78 (-19.59, -2.28)	0.01	-10.71 (-19.48, -2.32)	0.02	-1.14 (-10.26, 7.97)	0.80			-4.32 (-16.83, 7.84)	0.49		
**Number of comorbidities (0)$**												
**1**	-9.66 (-22.58, 2.62)	0.13	-17.32 (-29.95, -5.63)	0.009	-6.32 (-18.76, 6.12)	0.32	-5.89 (-18.13, 6.34)	0.36	-22.96 (-40.51, -6.03)	0.01	-19.93 (-36.47, -4.05)	0.02
**2**	-13.45 (-26.87, -0.69)	0.04	-14.96 (-28.29, -2.676)	0.03	-13.17 (-26.14, -0.18)	0.05	-15.53 (-27.99, -3.06)	0.02	-19.00 (-37.23, -1.43)	0.04	-19.16 (-36.56, -2.71)	0.03
**3 or more**	-14.78 (-30.54, 0.46)	0.06	-17.62 (-33.01, -2.97)	0.03	-17.36 (-33.09 -1.62)	0.03	-19.66 (-34.92, -4.41)	0.01	-26.47 (-47.96, -5.51)	0.01	-33.27 (-53.11, -14.20)	0.001

*Adjusted for clustering of patients within hemodialysis centers

**Table 4:** Health related quality of life of patients treated with hemodialysis from different studies using the KDQOL-36^™^

	Country				
	**Rwanda** ^**#**^	**Kenya**(28)	**Saudi Arabia**(30)	**USA**(31)	**USA**(32)
PCS	37.33	39.09	37.4	38	36.6
MCS	44.74	41.87	43.5	51.8	49.0
BKD	20.01	16.15	31.5	53.2	51.3
EKD	53.48	67.63	56.5	76.6	78.1
SPKD	58.22	73.46	74	80.7	73.0

**PCS** physical component summary, **MCS** mental component summary, **BKD** burden of kidney disease component summary, **EKD** effect of kidney disease component summary, **SPKD** symptoms and problem of kidney disease component summary **USA** United States of America #present study

## Data Availability

The datasets generated and analyzed during the current study are not publicly available because we are not allowed to share individual level data. However additional information about the data is available from the corresponding author on reasonable request.

## Declarations

All study participants signed written informed consent prior to study enrollment.
